# The impact of therapy for childhood acute lymphoblastic leukaemia on intelligence quotients; results of the risk-stratified randomized central nervous system treatment trial MRC UKALL XI

**DOI:** 10.1186/1756-8722-4-42

**Published:** 2011-10-13

**Authors:** Christina Halsey, Georgina Buck, Sue Richards, Faraneh Vargha-Khadem, Frank Hill, Brenda Gibson

**Affiliations:** 1 Department of Haematology, The Royal Hospital for Sick Children, Dalnair Street, Glasgow G3 8SJ, UK; 2 Institute of Infection, Immunity & Inflammation, College of Medical, Veterinary and Life Sciences, University of Glasgow, 120, University Place, Glasgow G12 8TA, UK; 3 Clinical Trial Service Unit, Richard Doll Building, Old Road Campus, Roosevelt Drive, Oxford, OX3 7LF, UK; 4 Developmental Cognitive Neuroscience Unit, UCL Institute of Child Health, 30 Guildford Street, London, WC1N 1EH, UK; 5 Department of Haematology, Birmingham Children's Hospital, Steelhouse Lane, Birmingham, B4 6NH, UK

**Keywords:** acute lymphoblastic leukaemia, IQ, central nervous system, morbidity, cranial radiotherapy, methotrexate, neuropsychometric, paediatric

## Abstract

**Background:**

The MRC UKALLXI trial tested the efficacy of different central nervous system (CNS) directed therapies in childhood acute lymphoblastic leukaemia (ALL). To evaluate morbidity 555/1826 randomised children underwent prospective psychological evaluations. Full Scale, verbal and performance IQs were measured at 5 months, 3 years and 5 years. Scores were compared in; (1) all patients (n = 555) versus related controls (n = 311), (2) low-risk children (presenting white cell count (WCC) < 50 × 10^9^/l) randomised to intrathecal methotrexate (n = 197) versus intrathecal and high-dose intravenous methotrexate (HDM) (n = 202), and (3) high-risk children (WCC ≥ 50 × 10^9^/l, age ≥ 2 years) randomised to HDM (n = 79) versus cranial irradiation (n = 77).

**Results:**

There were no significant differences in IQ scores between the treatment arms in either low- or high-risk groups. Despite similar scores at baseline, results at 3 and 5 years showed a significant reduction of between 3.6 and 7.3 points in all three IQ scores in all patient groups compared to controls (P < 0.002) with a higher proportion of children with IQs < 80 in the patient groups (13% vs. 5% at 3 years p = 0.003).

**Conclusion:**

Children with ALL are at risk of CNS morbidity, regardless of the mode of CNS-directed therapy. Further work needs to identify individuals at high-risk of adverse CNS outcomes.

**Trial registration:**

ISRCTN: ISRCTN16757172

## Background

Advances in the treatment of paediatric acute lymphoblastic leukaemia (ALL) have resulted in 5 year event-free survival rates of over 80% [1]. With such good survival, efforts are now focused on minimising treatment-related morbidity. One area of concern is the possible long-term effects of central nervous system (CNS) directed therapy on children.

Whilst CNS-directed treatments result in few long-term neurocognitive impairments in adults [2], they may adversely affect children whose neurocognitive systems are still in the process of maturing [3]. The first reports of adverse neuropsychological outcomes emerged in the 1970s and 80s after the introduction of universal CNS directed therapy - usually in the form of cranial irradiation (XRT) [4,5]. These initial observations led to attempts to identify the causative agents, any additional risk factors and the exact nature of the impairment. There followed numerous studies examining neurocognitive outcomes after various forms of CNS-directed treatment (for recent reviews see [6,7]) but drawing definitive conclusions from these studies is compromised by small patient numbers, differences in study design, the vast range of tests employed, use of historical cohorts, lack of proper control groups, non-random assignment of different CNS-directed treatments and changes in accompanying systemic therapy and supportive care over time [8,9].

Debate still exists over the most important causative agents, and in particular the relative impact of different CNS-directed treatments on neuropsychological outcomes. Early studies using global measures of intellectual functioning, such as intelligence quotients (IQs) and academic attainment, showed fairly consistent declines in patients treated with XRT [5,10-13]. This led to increasing avoidance of radiotherapy in many treatment protocols and, as a result, recent data are sparse. The outcome with chemotherapy-only regimens is more variable with some showing almost normal cognitive functioning [14-18], and others reporting reduced IQs [19]. A large meta-analysis [20] suggests chemotherapy alone is associated with modest declines in IQ and other neurocognitive functions. The relative impact of intrathecal methotrexate (IT MTX) versus high-dose systemic methotrexate (HDM) on CNS morbidity remains an important unanswered question especially since their equivalence in terms of overall survival means that any adverse side-effects are increasingly important.

An emerging view is that the mode of CNS-directed therapy may have little influence on adverse outcomes which may instead reflect the impact of the underlying disease and/or global manifestations of treatment: Two meta-analyses confining analysis to neuropsychological outcomes in studies which included valid control groups have shown that patients with ALL fare worse than controls regardless of their mode of CNS-directed therapy [20,21]. The choice of control group is vital since IQ is highly correlated with socioeconomic status [22] making comparison with population means inappropriate in most small to medium scale studies. Until now a sufficiently large randomised trial including an appropriate control group has been lacking to definitively address this question.

If the mode of treatment is not the main determinant of adverse outcome then the search for additional risk factors becomes even more important. A number of small studies have identified younger age [4,10,15,23,24] and female sex [10,25] as likely candidates. In an early meta-analysis [5], an age of 5 years or under at initial diagnosis was a significant factor but this study did not examine gender differences. Girls may fare worse, particularly in some areas such as verbal IQ [26] but existing meta-analyses are not sufficiently powered to answer this question [20]. Moreover, age and gender factors may interact to give rise to adverse outcome.

Against this background, the MRC UKALLXI psychometric study aimed to compare prospectively the neurocognitive effects of three different types of CNS-directed therapy (HDM vs. IT MTX and HDM vs. XRT). The study of a large cohort of children randomly allocated to different treatment regimens, and comparison with an appropriate control group allowed this study to address a number of important questions not yet reliably answered in the literature i.e. 1) In modern treatment protocols (with avoidance of radiotherapy in children under 2 years of age) is the use of cranial irradiation still associated with adverse neuropsychometric outcomes compared to high dose methotrexate? 2) Is high dose systemic methotrexate associated with different psychometric outcomes compared to intrathecal methotrexate? 3) Does age or gender influence susceptibility to adverse psychometric outcomes? 4) Can a subset of children at high risk of neurological adverse outcomes be identified to enable targeted intervention? 5) Is treatment for ALL associated with reduction in IQ test scores in patients compared to scores in age matched relatives?

No differences in event free survival (EFS) were seen between the two randomised treatment arms [27], thus increasing the importance of identifying any adverse effects of treatment. Here, we report the results of intelligence tests for patients and controls assessed at baseline and at 3 and 5 year time-points after initiation of treatment.

## Results

**Figure 1:**
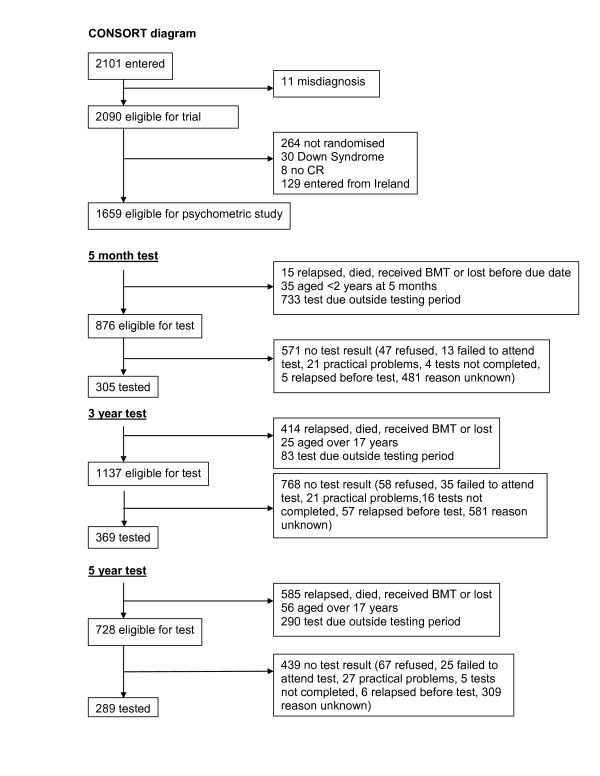
**CONSORT Diagram**.

During the study period 866 children had an IQ test (555 patients; 311 controls). As shown in the accompanying CONSORT diagram (Figure 1) the numbers of eligible patients tested at the three time points were 305/876 (35%), 369/1137 (32%) and 289/728 (39%), respectively. Thus, the proportion tested did not decrease as a function of time from diagnosis. Psychologists were asked to give priority to testing the high risk group, and this is reflected in the proportion of tests done (65% high risk, 30% low risk). Although a small proportion of the eligible patients were not tested due to specific reasons such as refusal, failure to attend for testing or practical problems (language, relocation, relapse prior to test etc.), the vast majority of eligible but untested patients were untested due to the time constraints of the psychologist's workload. There were no differences between randomised arms in the proportions tested.

Further information on the tests employed, standardisation of tests, statistical power calculations and choice of controls is detailed at the end of the paper.

### Patients versus controls

There were no significant differences in Verbal (VIQ), Performance (PIQ), or Full Scale IQ (FSIQ) scores between patients and controls at baseline (i.e. the 5 month test). However, clear differences were seen at 3 and 5 years in all three IQ scores (Table 1).

**Table 1:** IQ scores of patients and controls at each time period

	Number tested		Mean adjusted IQ* (SD)		Difference in means (95% CI)	t-test p-value
		
	Controls	Patients	Controls	Patients		
**FSIQ**						

5 months	157	284	102.5 (14)	101.0 (15)	1.4 (-1.5 : 4.4)	0.3

3 years	173	366	104.8 (14)	97.7 (16)	7.1 (4.4 : 9.8)	< 0.0001

5 years	132	289	105.2 (15)	100.0 (16)	5.2 (1.9 : 8.5)	0.002

**VIQ**						

5 months	158	287	102.0 (14)	99.6 (15)	2.3 (-0.5 : 5.2)	0.1

3 years	173	366	103.4 (15)	97.7 (15)	5.7 (3.1 : 8.4)	< 0.0001

5 years	132	289	103.2 (14)	99.6 (15)	3.6 (0.5 : 6.7)	0.02

**PIQ**						

5 months	160	299	102.5 (15)	102.0 (16)	0.5 (-2.5 : 3.5)	0.7

3 years	173	368	105.5 (15)	98.2 (16)	7.3 (4.5 : 10.1)	< 0.0001

5 years	132	289	106.4 (16)	100.5 (17)	5.9 (2.5 : 9.3)	0.0006

*IQ adjusted for test by subtracting:

7.15 from each WPPSI-R full IQ

3.79 from each WPPSI-R verbal IQ.

8.74 from each WPPSI-R performance IQ.

To explore this observed difference in IQ between patients and controls further, we examined the proportion of individuals with IQ scores lower than 80, since an IQ at this level would be expected to be functionally significant. At 5 months (baseline) there was no statistically significant difference in the proportion of patients with IQ scores (FSIQ, VIQ or PIQ) below 80. At 3 years 13% of patients and 5% of controls had FSIQ scores less than 80 (p = 0.003), with smaller but still significant differences in the proportions with FSIQ < 80 at 5 years (11% vs. 5% p = 0.03).

### Treatment Comparisons: Low Risk Group (HDM/IT MTX versus IT MTX)

The mean differences in FSIQ, PIQ or VIQ between patients randomised to HDM and IT MTX and those randomised to IT MTX alone were small, and non-significant, at 3 years and at 5 years (table 2), with confidence intervals ruling out 6 point differences. These results remain unchanged after allowing for age at the start of treatment, gender and number of previous tests taken. In addition, examining the proportion of patients with an IQ < 80 showed no differences by treatment allocation (data not shown).

**Table 2:** IQ in low risk randomisation groups (HDM versus intrathecal MTX)

	Number of patients		Mean adjusted IQ* (SD)		Difference in means (95% CI)	t-test p-value
		
	HDM	IT MTX	HDM	IT MTX		
**FSIQ**						

3 years	138	132	97.9 (16)	98.3 (16)	-0.4 (-4.4 : 3.5)	0.8

5 years	116	104	99.5 (15)	100.9 (18)	-1.4 (-5.8 : 3.0)	0.5

**VIQ**						

3 years	138	132	97.8 (15)	98.2 (15)	-0.4 (-4.0 : 3.3)	0.8

5 years	116	104	99.2 (14)	100.3 (17)	-1.1 (-5.2 : 3.0)	0.6

**PIQ**						

3 years	138	132	98.1 (17)	99.0 (17)	-1.0 (-5.0 : 3.1)	0.6

5 years	116	104	100.2 (15)	101.3 (19)	-1.1 (-5.7 : 3.4)	0.6

*IQ adjusted for test by subtracting:

7.15 from each WPPSI-R full IQ.

3.79 from each WPPSI-R verbal IQ.

8.74 from each WPPSI-R performance IQ.

### Treatment Comparisons: High Risk Group (HDM/IT MTX versus short course IT MTX/XRT)

As shown in table 3 there were no significant differences in FSIQ, PIQ or VIQ between patients randomised to high dose methotrexate and those randomised to cranial irradiation at 3 years or at 5 years, but the mean differences were somewhat larger in this group, and confidence intervals are wide (due to the smaller numbers tested) and can only rule out differences of 10 points. These results were unchanged when allowing for age at the start of treatment, gender, and the number of previous tests taken. Again, analysis of the proportion of patients with a FSIQ < 80 showed no difference by treatment allocation (data not shown).

**Table 3:** IQ in high risk randomisation groups (HDM versus XRT)

	Number of patients		Mean adjusted IQ* (SD)		Difference in means (95% CI)	t-test p-value
		
	HDM	XRT	HDM	XRT		
**FSIQ**						

3 years	45	51	98.9 (13)	94.7 (13)	4.2 (-1.1 : 9.4)	0.1

5 years	35	34	100.5 (16)	98.2 (15)	2.3 (-5.1 : 9.8)	0.5

**VIQ**						

3 years	45	51	98.9 (14)	94.9 (13)	4.0 (-1.5 : 9.5)	0.2

5 years	35	34	100.3 (16)	98.2 (15)	2.0 (-5.5 : 9.6)	0.6

**PIQ**						

3 years	45	51	99.4 (13)	95.7 (13)	3.7 (-1.5 : 8.8)	0.2

5 years	35	34	101.0 (15)	98.4 (14)	2.7 (-4.3 : 9.7)	0.4

* IQ adjusted for test by subtracting

7.15 from each WPPSI-R full IQ.

3.79 from each WPPSI-R verbal IQ.

8.74 from each WPPSI-R performance IQ.

### Effects of age and gender

As shown in Table 4 there was no evidence of differential effects on mean IQ scores between those aged under 5 years at the start of treatment and those aged 5 years and above. This was true for all 3 comparison groups - controls versus patients, IT MTX vs. HDM and HDM vs. XRT. Using the measure of IQ < 80, we also looked at the effect of age on the proportion of low-functioning individuals. By this criterion those aged < 5 years at the start of treatment were more likely to have a FSIQ < 80 at their 3 year test point than those aged > 5 years (17% vs. 7% respectively, P = 0.005).

**Table 4:** Effect of age and gender on mean difference in FSIQ

	Difference in mean FSIQ (95% CI)		p-value	Difference in mean FSIQ (95% CI)		p-value
				
	Age < 5	Age > 5		Male	Female	
**Controls vs. patients**						

3 years	7.7 (3.7: 11.7)	5.0 (1.2: 8.8)	ns	8.3 (4.5: 12.1)	5.6 (1.7: 9.5)	ns

5 years	5.6 (1.3: 9.9)	3.6 (-1.5: 8.7)	ns	5.5 (1.1: 9.9)	4.6 (-0.4: 9.6)	ns

**HDM vs. IT MTX**						

3 years	-0.1 (-4.9: 4.6)	1.0 (-5.7: 7.7)	ns	-0.9 (-6.4: 4.5)	0.0 (-5.7: 5.8)	ns

5 years	1.6 (-3.5: 6.7)	-6.6 (-14.5: 1.3)	ns	-1.3 (-7.6: 5.0)	-1.7 (-8.0: 4.6)	ns

**HDM vs. XRT**						

3 years	4.2 (-3.4: 11.9)	4.2 (-3.5: 11.8)	ns	3.6 (-3.8: 11.0)	4.8 (-3.1: 12.6)	ns

5 years	5.5 (-5.9: 13.4)	-0.4 (-10.8: 10.0)	ns	2.1 (-7.0: 11.2)	1.5 (-12.2: 15.2)	ns

The effect of gender on IQ was examined by multiple regression analysis. No statistically significant differences were seen between mean IQ scores in male and female patients in any of the groups.

There was no effect of gender on the proportion of patients with an IQ < 80 (data not shown).

## Discussion

We present here the largest study of neuropsychological outcomes in children treated for ALL. In addition to patient numbers, this study benefits from being randomized with respect to treatment regimes, a prospective design, and the inclusion of a control group of healthy children. Despite the recognised problems of using different tests and standardizations for different age groups this study has produced clear results.

Firstly, there were no significant differences between patients randomised to continuing intrathecal methotrexate alone compared with those randomised to additional high dose methotrexate. This was true for both the under, and the over 5-year old age groups, and for both sexes. The numbers of participants in these comparisons were large allowing reasonable confidence that important differences do not exist. These findings are consistent with the majority of smaller studies [14-18] and meta-analyses [20] in the literature.

Similarly, we found no significant differences in IQ scores in those randomised to cranial irradiation compared with those randomised to high dose methotrexate. Although possibly unexpected, our results mirror those of another recent study showing that with modern protocols the neuropsychological outcomes for XRT and chemotherapy-only groups are very similar [28]. Importantly, the UKALL XI protocols used a relatively high dose of cranial irradiation (24 Gy) further strengthening results of Waber [28] whose protocols only used 18 Gy. In addition, relatively early folinic acid rescue (commencing 36 hours after the start of the HDM infusion) may have reduced late effects of HDM. Both of these factors would have been expected to widen any gap between HDM and XRT in terms of adverse effects. These results contrast with earlier reports of significant impacts of cranial irradiation on IQ and other measures of intellectual functioning [4,13,14,18,24,29]. Several possibilities may explain these discrepant results. Firstly, the majority of studies showing adverse effects of cranial radiotherapy included very young children, and in many the adverse effects of radiotherapy were strongly associated with the younger age groups [10,14,18,24,25,29,30]. Since radiotherapy is thought to cause neurotoxicity predominantly by demyelination [31] and myelination is not complete until much later in childhood, younger children would be expected to be particularly vulnerable. Our study avoided all radiotherapy in children under 2 years of age and in addition the XRT randomisation was confined to children with a WCC > 50 - a biological feature associated with older age. Secondly, the use of an adequate control group is vital since studies that showed a detrimental effect of radiotherapy may have been demonstrating a detrimental effect of ALL and its treatment rather than a specific effect of XRT alone [5]. This is supported by carefully controlled longitudinal studies from the St Jude group which showed no difference between XRT and chemotherapy groups at a single time point [32], but subsequent longitudinal follow-up showed a decline in both treatment groups over time [26,33]. Thirdly, most reports of XRT effects pre-date the current treatment era and therefore changes in accompanying systemic therapy, supportive care or improved delivery methods may have either reduced the morbidity from cranial radiotherapy or narrowed the gap by increased neurotoxicity with intensified systemic therapy. This is supported by data from animal models [34] and patients [35] suggesting that systemic chemotherapy can have synergistic or protective effects when combined with XRT. Finally, the majority of previous reports involved non-randomised, retrospective studies of small numbers of patients and may have therefore been inadvertently biased towards recruitment of children with poorer outcomes and/or publication bias.

Although overall our data do not suggest that age is a significant risk factor for mean IQ values, children aged less than 5 years at initial diagnosis are more likely to have IQ below 80 at 3 years compared to children aged over 5 years at diagnosis, irrespective of treatment allocation. This is consistent with models of brain development suggesting that younger children are likely to be particularly vulnerable to neurotoxic insults. These data also highlight that mean IQ values may mask significant individual declines in IQ as discussed below.

The finding of similar outcomes in males and females is reassuring. Initial reports of inferior outcomes in girls came from relatively small studies using combinations of methotrexate and cranial radiotherapy [10,25]. More recently a number of chemotherapy-only protocols have also shown inferior outcomes in girls [17], although a meta-analysis of chemotherapy-only protocols could not reach a firm conclusion [20]. The possible underlying mechanisms for gender differences in neuropsychological outcome in ALL are poorly understood and in fact in other areas of acute brain injury, such as head injury, girls usually have better outcomes than boys. Again, changes in therapy protocols such as lack of co-administration of high-dose methotrexate and avoidance of radiotherapy in young patients may explain the lack of difference in IQ in our studies.

Importantly, despite the lack of effect of randomised treatment allocation on IQ, patients definitely fared worse than controls, with a lower mean IQ of between 5 and 7 points. The effect was seen for FSIQ, VIQ and PIQ. A reduction in IQ score of this magnitude may be of only modest impact in children with average or above average initial IQ scores but importantly this effect also translates into a larger proportion of children with IQ scores less than 80 - a level consistent with low intellectual functioning. These results suggest that children treated for ALL are at risk of neurodevelopmental morbidity regardless of which of these randomised CNS-directed therapies they received. This has previously been suggested by smaller studies [33,36], a meta-analysis [21], and a recent larger study [28] which reported some selective weaknesses in verbal IQ and mathematics fluency in all children with ALL regardless of their treatment allocation. It is also supported by a lack of dose response for both radiotherapy [26] (18 Gy vs. 24 Gy) and methotrexate [18] (HDM vs. very high dose MTX). It is known that even intrathecal methotrexate alone can be associated with white matter changes, calcifications, leukoencephalopathy, cortical atrophy, and seizures in some patients [37].

Some important limitations of our study should be acknowledged. A cross-sectional design was necessary to maximise patient recruitment in order to answer the main study questions but this design makes it impossible to track the unfolding of impairments in individual patients over time. The large numbers of participants, balanced randomisation and inclusion of a socioeconomically matched control group makes substantial demographic differences in the tested cohorts in the different arms at 3 time-points unlikely, but some alteration in the demography of the groups over time cannot be excluded, and it is possible that this may explain improvement in scores at 5 years. Secondly, IQ tests are a relatively global measure of intelligence. A multitude of more specific defects have been reported in the literature with a particular propensity for domains such as attention, arithmetic fluency, non-verbal reasoning, to be affected [13,15,38]. We chose to investigate IQ as a primary outcome measure because it was so well standardised and relatively robust but these results do not exclude the possibility of specific influences of our randomised treatment arms on more subtle but important neuropsychological measures. Whilst investigation of these additional measures would obviously add to our findings it does not detract from our major observation of a difference in mean IQ between patients and controls.

Overall these data suggest that factors other than the mode of CNS directed treatment determine the likelihood of CNS morbidity and that there may be vulnerable groups of children who manifest large declines in IQ whilst others are relatively unaffected. That mean IQ scores comfortably fall in the average range will be a huge reassurance to most parents and patients - attention now needs to be focussed on identifying the smaller subset of vulnerable children. Study of these children (alongside matched unaffected controls) should allow identification of possible risk-factors. Candidates include; inherent genetic susceptibility, drug toxicity, time out of full-time education or particular vulnerability of certain individuals to the impact of chronic illness. Pharmacogenomic and genome wide association studies comparing severely affected children with those with persistently normal IQs should help identify genetic and drug-related risk factors. Indeed a recent report implicates polymorphisms in folate metabolism pathways as a risk factor for CNS morbidity [39]. Correlative neuro-imaging may also help identify aetiology, as it is possible to quantify leukoencephalopathy using MRI [40] and functional MRI offers an exciting new approach [41]. Systemic drugs used in all children with ALL include anti-folates, steroids and nucleoside analogues all of which have documented neurotoxic side effects [30,42,43]. The equivalent results in pre-school and older children argue against frequent and/or prolonged absence from school being the primary cause for the observed reduction in IQ.

## Conclusions

In summary, with modern protocols and avoidance of XRT for very young children, the neuropsychological outcomes for XRT and chemotherapy-only groups are very similar. We are unable to confirm female gender as a risk factor, but children aged below 5 years may be more vulnerable to treatment related neurotoxic effects. The most striking finding of this study is the difference observed between patients and controls, regardless of the CNS treatment delivered. This supports the view that ALL itself, and the necessity for intensive treatment, has a detrimental effect on IQ in some children. Detailed longitudinal neuropsychological assessments should allow individualised risk factors for neurocognitive morbidity to be examined. We predict that improvements in neuropsychological outcomes for children with ALL will depend more on individualised therapy for children at high risk of CNS morbidity than on avoidance of specific CNS-directed therapy regimens in unselected patient cohorts.

## Patients and Methods

### The UKALLXI Trial

Between 1990 and 1997 a total of 2090 patients with ALL entered UKALLXI, with 1826 randomized for CNS-directed therapy. Low-risk children (presenting WBC < 50 × 10^9^/l) (n = 1513) were randomized between intrathecal methotrexate alone (IT MTX) or in combination with high dose intravenous methotrexate (HDM) (8 g/m^2 ^for those below 4 years of age and 6 g/m^2 ^for those aged 4 years or above, folinic acid rescue commenced at 24 hours). High-risk children (presenting WBC of ≥ 50 × 10^9^/l) (n = 313) were randomized to receive HDM and continuing IT MTX or a short course of IT MTX followed by cranial irradiation (XRT) (2400 Gy), with the exception of those under the age of 2 years who were all allocated HDM. The 26 children with overt CNS disease were treated with cranial radiotherapy and excluded from this study. For details of the full treatment regimen see Table 5. There were no significant differences in event-free survival by treatment allocation [27].

**Table 5:** UKALL XI treatment regimen

Induction	Vincristine 1·5 mg/m^2 ^i.v. days 1, 7, 14, 21
Weeks 1-4	Prednisolone 40 mg/m^2 ^p.o. days 1-28
	L-Asparaginase 6000 U/m^2 ^s.c./i.m. t.i.w. nine doses
	IT MTX days 1, 8
Intensification	Vincristine 1·5 mg/m^2 ^i.v. day 1
Weeks 5-7	Prednisolone 40 mg/m^2 ^p.o. days 1-7 then 7 d taper
	Etoposide 100 mg/m^2 ^i.v. days 1-5
	Cytarabine 100 mg/m^2 ^i.v. given 12 hourly days 1-5
	Daunorubicin 45 mg/m^2 ^days 1, 2
	Thioguanine 80 mg/m^2 ^p.o. days 1-5
	IT MTX day 1

Intensification	Vincristine 1·5 mg/m^2 ^i.v. day 1
Weeks 20-22	Prednisolone 40 mg/m^2 ^p.o. days 1-5
	Etoposide 100 mg/m^2 ^i.v. days 1-5
	Cytarabine 100 mg/m^2 ^i.v. given 12 hourly days 1-5
	Daunorubicin 45 mg/m^2 ^days 1, 2
	Thioguanine 80 mg/m^2 ^p.o. days 1-5
	IT MTX day 1

CNS-directed treatment weeks 8-19: Randomization WBC ≤ 50 × 10^9^/l	IT MTX weekly (weeks 9-12) or HDM 6 g/m^2 ^(≥ 4 years old) or 8 g/m^2 ^(< 4 years old) weeks 9, 11, 13 + IT MTX weeks 9, 11, 13, 14. HDM IV over 24 hours, folinic acid rescue commenced at 36 hours from start at 15 g/m^2 ^3-hourly, reduced to 15 g/m^2 ^6-hourly once serum MTX level < 2 × 10^6 ^mol/l and stopped once serum MTX level below 1 × 10^7 ^mol/l.

CNS-directed treatment weeks 8-19: Randomization WBC ≥ 50 × 10^9^/l	HDM + IT MTX as above or 24 Gy cranial radiotherapy in 15 fractions of 1·6 Gy each in weeks 9-12 (except children of 1-2 years age who were allocated HDM)
	Plus IT MTX weeks 9-11

Interim continuation therapy	Mercaptopurine 75 mg/m^2 ^p.o. daily
Weeks 8-19	Methotrexate 20 mg/m^2 ^p.o. weekly except when ITMTX given
and 23-34	Vincristine 1·5 mg/m^2 ^i.v. every 4 weeks
	Prednisolone 40 mg/m^2 ^p.o. daily × 5 d every 4 weeks.

Continuation therapy Weeks	Same as above ± 3-monthly ITMTX
35 or 43-100	Age-adjusted

Third intensification Weeks 35-42	Dexamethasone 10 mg/m^2 ^p.o. for 10 d then 4 d taper
	Vincristine 1·5 mg/m^2 ^i.v. days 1, 7, 14, 21
	L-Asparaginase 6000 U/m^2 ^s.c./i.m. t.i.w. nine doses
	IT MTX (age-adjusted) days 1, 28
	Cyclophosphamide 600 mg/m^2 ^i.v. days 28, 42
	Cytarabine 75 mg/m^2 ^i.v./s.c. days 28-31, 35-38, 42-45, 49-52
	Thioguanine 60 mg/m^2 ^p.o. days 28-56

1. IT MTX, intrathecal methotrexate; HDM, high-dose intravenous methotrexate; t.i.w, given on alternate days for three days each week.

### The UKALL XI Neuropsychological Study

All UKALLXI randomised patients aged between 2 and 16 years were eligible for the Neuropsychological study except children with Down syndrome, or those who had relapsed or undergone bone marrow transplantation.

Where possible, one healthy related control was recruited for each index patient. Relatives were chosen as controls to ensure reasonable matching for socioeconomic status and disruption to normal family life and because IQ is generally well correlated between siblings [44]. Where more than one potential control was available they were selected by closest age, followed by gender. If no sibling control was available, cousins (of similar age and/or gender) were invited to participate. Lack of a suitable control did not exclude a patient from the study.

Neuropsychological tests were administered at 5 months, 3 years and 5 years from the start of treatment for patients, and at comparable intervals for their controls. Some flexibility was allowed around the ideal test date: Within the first year for the 5 month test, and 1 year either side of both the 3- and 5-year test dates. The study was not designed as a longitudinal study, but rather as a cross-sectional prospective study, in order to maximise the number of follow-up tests completed (at 3 and 5 years) by patients within the period of funding. Thus the neuropsychological study did not commence until 2 years into the UKALL XI trial and preference was always given to 3 and 5 year tests over 5 month tests if a choice had to be made.

Table 6 summarises the numbers of children tested in each category and time point. There were no significant differences in age, time of testing, or gender by randomised treatment allocation. Controls were older, with a median age of 6 years for controls and 4 years for patients (p < 0.001) and tested at a median of 1-2 months later (p < 0.005) than patients.

**Table 6:** Numbers assessed at each time period in each treatment group

	Control	Patient				
		
		Any	High Risk		Low Risk	
				
			XRT	HDM	HDM	IT MTX
Any test	311	555	77	79	202	197
5 month test	161	305	47	42	104	112
3 year test	173	369	51	45	139	134
5 year test	132	289	34	35	116	104
						
5 month only	92	133	23	28	35	47
3 year only	57	94	15	16	34	29
5 year only	37	40	3	6	21	10
5 month and 3 year only	30	39	5	0	17	17
5 month and 5 year only	9	13	0	0	7	6
3 year and 5 year only	56	116	12	15	43	46
All 3 tests	30	120	19	14	45	42

### Neuropsychological assessment

Three standardized scales were used to evaluate intellectual ability (IQ): Children aged ≥ 2 to < 6 years were assessed on the Wechsler Preschool and Primary Scale of Intelligence - Revised (WPPSI-R); children aged ≥ 6 to < 17 years on the Wechsler Intelligence Scale for Children - 3^rd ^Edition UK (WISC-III); and those aged ≥ 17 years and above on the Wechsler Adult Intelligence - Revised Scale (WAIS-R). Scaled subtest scores were summed to obtain estimates of Full Scale IQ (FSIQ), Verbal IQ (VIQ), and Performance IQ (PIQ). All IQ scores are standardized (mean = 100, standard deviation = 15).

The majority of children initially assessed on the WPPSI-R scale moved on to the WISC-III scale at their 3 year or 5 year test points (as they entered the 6-16 age range). Changes in the assessment tool can produce an apparent drop in IQ over time [8,26,45,46], and therefore it was important to carefully consider their equivalence. Analysis of results from the first test taken by controls (n = 311) showed that WPPSI-R scores were higher than WISC-III scores for FSIQ (difference 7.15: p < 0.0001), VIQ (difference 3.79: p = 0.04), and PIQ (difference 8.74: p < 0.0001) (Table 7). Due to these large differences, all WPPSI-R test scores were adjusted downwards by subtraction of 7.15, 3.79 and 8.74 from FSIQ, VIQ and PIQ scores respectively. These adjusted IQ scores were used for subsequent analysis. Where possible, results were validated by allowing for "type of test" (WISC-III, WPPSI-R or WAIS-R) as a covariate in a multiple regression model.

**Table 7:** First IQ score by test type: Controls only

	First test			WPPSI-R v WISC-III	
	**WAIS-R** **(n = 9)**	**WPPSI-R** **(n = 87)**	**WISC-III** **(n = 215)**	**Difference** **in IQ**	**t-test** **p-value**

FSIQ (mean) (std dev)	104.00 (13.6) n = 9	109.75 (14.0) n = 84	102.60 (13.6) n = 214	7.15	< 0.0001

VIQ (mean) (std dev)	100.33 (14.7) n = 9	106.08 (13.6) n = 84	102.30 (14.0) n = 215	3.79	0.04

PIQ (mean) (std dev)	108.33 (13.4) n = 9	111.17 (14.6) n = 87	102.43 (14.3) n = 214	8.74	< 0.0001

### Practice effects over time

Although IQ scores in an individual are generally stable over time, there are reported increases of 7-8 points in FSIQ score if the re-test interval is short. An interval of 6-12 months is reportedly sufficient to nullify these so called practice effects [47]. Practice effects are different for VIQ and PIQ; very low in the case of the former, but much higher in the case of the latter.

Analysis of the IQ scores in our control group suggests the presence of a practice effect. Out of 132 controls tested at the 5 year time point, 37 were taking their first test, 65 their second and 30 their third. The corresponding FSIQ means were 101, 106 and 109 respectively. A one-way analysis of variance exploring the 5 year FSIQ by the number of previous tests taken yielded a p-value of p = 0.02. For the 3-year tests, controls taking their second test had a mean FSIQ of 107 (n = 60), compared to a mean of 103 (n = 113) in those previously untested (p = 0.08). As a result of these findings, the number of previous tests performed was included as a covariate in multiple regression models.

Finally, IQ test scores have increased over the years (the Flynn effect) [48]. Examination of the controls' data sets failed to show any time-related changes. Since the study duration was short, this effect was not considered further.

### Statistics

Since intelligence scores are normally distributed, t-tests were employed for these analyses, and multiple regression methods (using the SAS procedure GLM) were used to validate these results, with the p-value for heterogeneity taken from the relevant interaction term. The Mann Whitney U-test (2 groups) and Wilcoxon's Rank Sum Test (multiple groups) were used for comparisons of non-normal scores. Gender by treatment group was investigated using the chi-square test - and Fisher's exact test when the expected numbers were small. All analyses were performed using the SAS statistical package.

The main aim was to compare the IQ scores of the randomised treatment groups at follow-up. Power calculations were based on estimated effect sizes from the largest meta-analysis available at the time [5]. The target number in the high-risk group was 112 patients tested at 3 years, to give 90% power to detect a difference of 9 points in the full IQ scores. The target number in the low-risk group was 438 patients tested at 3 years, giving over 95% power to detect a difference of 4 points in the full IQ score. Further power calculations were performed to estimate required sample numbers for subgroup analysis of the effect of age on IQ with 56 patients in each group required to give an 80% chance of detecting a difference of 10 IQ points in the high risk group, and 219 patients in each group required to give an 85% chance of detecting a difference of 4 IQ points in the low risk group.

### Ethical Approval

Individual centres in the UK obtained ethical approval from their local research ethics committee and obtained informed consent from parents and patients (where appropriate for age) before entering patients into the study.

## List of Abbreviations

ALL: Acute lymphoblastic leukaemia; CNS: Central nervous system; IQ: Intelligence Quotient; MTX: methotrexate; WCC: white cell count; IT: intrathecal; HDM: High dose methotrexate; XRT: Radiotherapy; EFS: Event free survival; VIQ: Verbal intelligence quotient; PIQ: Performance intelligence quotient; FSIQ: Full scale intelligence quotient; MRC: Medical Research Council (UK).

## Competing interests

The authors declare that they have no competing interests.

## Authors' contributions

BG, FH, F V-K and SR designed the research study, GB, SR, BG and CH analysed the data, CH, BG, GB and SR wrote the paper. All authors read and approved the final manuscript.
